# Differences in the length of postoperative care of endoscopic endonasal surgery in simple and complex surgical procedure groups

**DOI:** 10.12688/f1000research.110037.1

**Published:** 2022-06-07

**Authors:** Budi Sutikno, Fuad Fauzi, Diar Mia Ardani, Anna Mailasari

**Affiliations:** 1 Department of Otorhinolaryngology, Head and Neck Surgery, Faculty of Medicine, Universitas Airlangga - Dr. Soetomo Academic Medical Center, Surabaya, East Java, 60286, Indonesia; 2 Department of Otorhinolaryngology, Head and Neck Surgery, Faculty of Medicine, Universitas Diponegoro - Dr. Kariadi General Hospital, Semarang, Central Java, 50275, Indonesia

**Keywords:** endoscopic endonasal surgery, length of care, postoperative, sinus disease

## Abstract

**Background:** The rapid development of endoscopic endonasal surgery has made the procedure widely used in nasal and sinus surgery. Endoscopic endonasal surgery is a minimally invasive procedure, but the possibility of postoperative damage to the sinonasal mucosa cannot be ruled out. The aim of this study was to analyze the difference in the length of postoperative care between complex and simple endoscopic endonasal surgeries which can be used as a reference in planning postoperative care.

**Methods:** This was a retrospective cross-sectional observational study. The participants were divided into two groups, i.e., simple, and complex surgical procedures groups. The simple procedure group consisted of middle meatal anstrostomy, uncinectomy, endoscopic septoplasty, and endoscopic turbinoplasty. The complex procedure group included frontal sinusotomy, sphenoidectomy, as well as anterior and posterior ethmoidectomy. The length of postoperative care between the two groups were measured and analyzed using the Chi-square test.

**Results: **The median length of care in the complex procedure group was significantly longer than that in the simple procedure group (p = 0.028), 12 weeks and 9 weeks, respectively. The number of postoperative outpatient visits was significantly less in the simple procedure group compared with the complex procedure group (Median 4 vs. 5; p=0.015). There was a significant correlation between length of care and the endonasal endoscopic surgical procedure group (p = 0.023).

**Conclusions: **The complex endoscopic endonasal surgery group required a longer length of care and more postoperative outpatient visit than the simple procedure group.

## Introduction

The endoscopic endonasal approach has become one of the technological advances in the otorhinolaryngology head and neck surgery (ORL-HNS) field, used as a supporting examination for diagnosis and therapy. In the 1970s, endoscopic endonasal and intranasal surgical interventions were first introduced by Messerklinger, Stammberger, Draf, and Wigand through transitioning sinus surgery from radical surgery to minimally invasive procedures. ^1^ Minimally invasive procedures such as endoscopic endonasal surgery are currently widely performed in the Ear, Nose & Throat (ENT-HN) field, including functional endoscopic sinus surgery (FESS), endoscopic turbinoplasty, and endoscopic septoplasty.

In the FESS procedure, ethmoid air cells and sinus ostia are opened by direct visualization aimed at restoring ventilation and mucociliary drainage of the sinus to normal function. ^1^ In recent decades, FESS has become the standard of care for chronic sinusitis, along with the developments in procedural knowledge. ^1^ The FESS procedure may include one and/or several different techniques, i.e., uncinectomy, Middle Meatal Antrostomy (MMA), anterior and posterior ethmoidectomy, sphenoidectomy, and frontal sinusotomy. ^2^

Based on the level of experience of the ENT-HN specialist at the Dr. Soetomo Academic Medical Center, Surabaya, endoscopic endonasal surgery is divided into simple and complex surgical procedures. The simple surgical procedure group includes uncinectomy, MMA, endoscopic turbinoplasty, and endoscopic septoplasty, while the complex surgical procedure group includes frontal sinusotomy, anterior and posterior ethmoidectomy, and sphenoidectomy.

Endoscopic endonasal surgery is a minimally invasive procedure, but postoperative injury such as damage to the sinonasal mucosa remains unavoidable. Proper management and follow-up are essential in a patient’s postoperative recovery and wound healing in terms of endoscopic endonasal surgery. ^3^ Postoperative care in clinical practice differs from one another due to preference based solely on the recommendations or opinions of specialists and the results of randomized controlled trials. ^4^ The study conducted by Mielcarek-Kutcha *et al*. reported that the length of postoperative care for endoscopic endonasal surgery ranged from six weeks to three months. ^5^ Studies on the length of postoperative care for endoscopic endonasal surgery are limited. Until recently, there haven’t been any studies on the difference in the length of postoperative care for endoscopic endonasal surgery at Dr. Soetomo Academic Medical Center in Surabaya. Thus, the clinicians inadequately provide information to patients who undergo endoscopic endonasal surgery.

Based on this reason, this study attempted to identify the difference in the length of postoperative care for complex and simple endoscopic endonasal surgery so that the results can be used as a reference in the planning of postoperative care.

## Methods

This was an analytic observational study with a retrospective cross-sectional design. The research data were obtained from medical records to determine the difference in the length of postoperative endoscopic endonasal care in the simple and complex surgical procedure groups.

In total, 60 patients who underwent endoscopic endonasal surgery at Dr. Soetomo Academic Medical Center, Surabaya, from 2015 to 2020, were included (See underlying data). ^6^

The inclusion criteria were male and female patients with minimum age of 18 years. The exclusion criteria were patients that had extended endoscopic sinus surgery (ESS), malignancy, non-standard therapy, and incomplete medical records. The data was collected through a special Data Collection Sheet in the form of tabulations, graphs, and text presentations explaining the graphs.

### Data analysis

Microsoft Excel and SPSS IBM version 22 software were employed to analyze the data with univariate analysis for demographic and clinical characteristics, as well as bivariate analysis of two related variables. For normally distributed data, t- test with free samples was performed. Mann-Whitney test was applied to the non-normal distributions. Meanwhile, the Chi-square test was carried out to identify the correlation between variables. P<0.05 was considered statistically significant.

### Ethical approval

Ethical approval (Number 0340/LOE/301.4.2/II/2021) was obtained from the Ethics Committee of Dr. Soetomo Academic Medical Center, Surabaya, Indonesia. Oral Informed consent was obtained from all individuals included in this study.

## Results

Although not statistically significant, the mean age in the simple surgical procedure group was lower than that in the complex surgical procedure group, (p=0.099). Additionally, statistical significance was also not observed in gender difference between the two groups (p=0.315). The results indicate that 56% of the simple surgical procedure group were male, and 57.1% of the complex surgical procedure group were female ( Table 1).

**Table 1.** Patient’s demographic data.

Variable	Types of surgical procedures in endoscopic endonasal surgery		p-value
	Simple (n=25)	Complex (n=35)	
Age (years), mean±SD	39.64±13.28	46.08±15.62	0.099 ^*^
Gender, n (%)			
Male	14 (56.0%)	15 (42.9%)	0.315 ^**^
Female	11 (44.0%)	20 (57.1%)	

^*^ The independent samples t-test; the result would be significant if the p-value was lower than 0.05.

^**^ The Chi-square test; the results would be significant if the p-value was lower than 0.05.

In the simple endoscopic endonasal group, 76% of the patients complained about nasal obstruction, while 74.3% of patients that had undergone complex surgical procedures had nasal discharge ( Table 2). Additionally, the main risk factors in 28% the patients that underwent the simple surgical procedures included smoking and allergies. On the contrary, allergies and gastroesophageal reflux were discovered in 25.7% of the patients in the simple surgical procedures group. The maximal medical treatment (MMT) history was discovered more in the patients who underwent the complex surgical procedures (37.1%), compared to the simple surgical procedures group, (20.0%).

**Table 2.** Characteristic of postoperative care patients.

Variable		Types of surgical procedures in endoscopic endonasal surgery			
		Simple (n=25)		Complex (n=35)	
		n	%	n	%
main complaints	nasal obstruction	19	76.0	25	71.4
	nasal discharge	14	56.0	26	74.3
	facial pain	18	72.0	23	65.7
	reduced smell	6	24.0	15	42.9
risk factors	allergies	7	28.0	9	25.7
	asthma	1	4.0	4	11.4
	gastroesophageal reflux	6	24.0	9	25.7
	smoking	7	28.0	6	17.1
MMT history	yes	5	20.0	13	37.1
past surgical history	yes	6	24.0	10	28.6
types of past surgical	FESS	1	4.0	0	0.0
	sinus surgery	3	12.0	3	8.6
	nasal polypectomy	2	8.0	4	11.4
	septoplasty	0	0.0	1	2.8
diagnosis	chronic Rhinosinusitis with polyp	2	5.7	14	40.0
	chronic rhinosinusitis without polyp	16	45.7	12	34.3
	deviated septum	8	22.9	9	25.7
	Inferior turbinate hypertrophy	6	17.1	3	8.6

As much as 28.6% of the patients in the complex surgical procedure group had past surgical history, which was higher than the 24% of the patients in the simple surgical procedures group. In addition, the most surgical history types in the simple surgical procedures group was sinus surgery/nasal irrigation (8.1%), however, in the complex surgical procedure group nasal polypectomy was the main surgical type (11.4%) ( Table 2).

The most diagnosed disease was Chronic Rhinosinusitis (CRS) without polyps in the simple surgical procedures group (45.7%), while CRS with polyps was the most identified in the complex surgical procedures group (40.0%).

The t-test results of the length of postoperative care and the number of visit variables of endoscopic endonasal postoperative patients at ENT-HN Outpatient Unit at Dr. Soetomo Academic medical center in Surabaya suggested that the number of visits was statistically significant in both groups (p=0.015) ( Table 3).

**Table 3.** Length of care and the number of postoperative visit.

Variable	Types of surgical procedures				p-value
	Simple (n=25)		Complex (n=35)		
	Median	Range	Median	Range	
length of postoperative care (week)	9	2-34	12	3-81	0.028 ^*^
number of visit (times)	4	1-9	5	2-27	0.015 ^*^

^*^ p=0.015.

Based on the analysis of the receiver operating characteristics (ROC) curve, the obtained cut-off point for the length of postoperative care in both groups was six weeks with an area under the curve of 0.667 (0.528-0.807) and a p-value of 0.028. In accordance with the cut-off-point value, the length of postoperative care was categorized into less than six weeks and more than six weeks, as listed in Table 4.

**Table 4.** The t-test of the length of Postoperative care for the endoscopic.

Types of surgical procedures in endoscopic endonasal surgery	Length of postoperative care		p-value	RR
	≤6 weeks	>6 weeks		
Simple	15 (60.0%)	10 (40.0%)	0.023 ^*^	2
Complex	5 (14.3%)	30 (85.7%)		

^*^ p=0.023.

According to the analysis employing the Chi-square test ( Table 4), there was a significant correlation between the endoscopic endonasal surgical procedure groups and the length of postoperative care with a cut-off point of six weeks (p=0.023). The complex endoscopic endonasal surgical procedure group required significantly longer postoperative care, compared to the simple surgical procedure group. The complex endoscopic endonasal surgical procedure group required more than six weeks for postoperative care, which is double the length of care in the simple surgical procedure group.

## Discussion

The median length of postoperative care for the complex surgical procedure group was 12 weeks with a range of 3-81 weeks, while for the simple surgical procedure group it was nine weeks with a range of 2-34 weeks. Additionally, the median length of postoperative care in the complex surgical procedure group was significantly longer than in the simple surgical procedure group. This study indicated a significant correlation between the length of postoperative care and the endoscopic endonasal surgical procedure groups. The complex endoscopic endonasal surgical procedure group required significantly longer postoperative care, approximately more than six weeks, than the simple surgical procedure group.

As reported by other studies, it is most likely that the length of postoperative patient care was influenced by the appropriate management and follow-up of endoscopic endonasal surgery. ^7^ Mielcarek-Kuchta *et al*. reported that the length of postoperative care for endoscopic endonasal surgery patients ranged from six weeks to three months. Meanwhile, Dursun *et al*. reported that the length of the postoperative care ranged from 2 to 54 months, with an average period of 23 months. Another study stated that postoperative care of 12 endoscopic endonasal surgery patients was conducted for 6 months. ^8^

Previous studies also mentioned that the length of postoperative follow-up, which might describe the length of postoperative care and the success of the surgery, was assessed by the percentage of recurrency and symptom resolution. ^9^ Another study conducted postoperative clinical follow-up on the type of procedure in the complex surgical procedure group based on the patient postoperative clinical condition, the endoscopic and CT scan findings, as well as the presence or absence of complications and revision surgery to assess the healing process and the success of the surgery. ^10^

Several studies indicated that the duration of the wound healing process in the simple surgical procedures were relatively shorter than that in the complex surgical procedures. For instance, when studying the uncinectomy procedure, Mekhiemer *et al.* stated that the wound healing process in partial uncinectomy was approximately one or three weeks compared to approximately two or three weeks in total uncinectomy. Also, Byun dan Lee discovered that the wound healing process ranged from 1.18 to 2.36 weeks in partial uncinectomy and from 1.63 to 3.21 weeks in total uncinectomy. ^11^

Wound healing in those studies was defined by complete closure of the uncinectomy site by normal mucosa. The aforementioned studies support the results of this study, which indicated that the complex surgical procedure group had a longer healing period than the simple surgical procedure group.

Patients who underwent FESS were monitored for a period of 4 to 14 months, with a median follow-up of nine months. Postoperative outcomes were evaluated subjectively and objectively. The objective evaluation consisted of nasal endoscopy and, if possible, CT evaluation. The objective evaluation criteria consisted of epithelialization of the healthy ethmoid cavity, no sign of pathological secretions, and adequate opening of the natural ostium. ^12^

During the primary follow-up, thorough endoscopic cleaning of the cavity and separation of developed adhesions was performed on the 5 ^th^ to 7 ^th^ postoperative days. Endoscopic inspection and removal of the remaining crusts were performed on the 14 ^th^ postoperative day. During the secondary follow-up, which was approximately three months post operation, an endoscopic inspection of the cavity was performed in all patient groups. However, in patients with polypoid disease this took place approximately six months post operation. The primary postoperative follow-up of FESS consisted of three or four visits during the first two or three weeks until the ethmoid healed. Blood clots, crusts, etc., were removed endoscopically, and nasal saline irrigation was administered. Intranasal corticosteroid was applied as previously described. ^13^

Frontal sinusotomy surgery patients in the complex surgical procedure group required a twice-weekly visit for the first two weeks post operation. The frontal recess was cleaned and irrigated sufficiently. The frontal sinuses could also be irrigated during the initial postoperative visit, especially if a stent is placed. Some postoperative patients experience transient frontal pain for a year or more, but they responded well to weekly saline irrigation of the sinuses for two or three weeks. ^14^

## Conclusions

Endoscopic endonasal surgery is a minimally invasive procedure, but the possibility of postoperative damage to the sinonasal mucosa cannot be ruled out. The complex endoscopic endonasal surgery group required a longer length of care and more postoperative outpatient visit than the simple procedure group. The goals of postoperative care are to promote wound healing and early mucosal regeneration, reduce local inflammation, and minimize symptoms in the early postoperative phase. Postoperative care is expected to improve the quality of life that is sustainable in the long term and can minimize the possibility of revision surgery. The limitation in this study is in determining the grouping of the simple and complex surgical procedure groups.

## Data availability

### Underlying data

Figshare: Differences in the length of postoperative care of endoscopic endonasal surgery in simple and complex surgical procedure groups 10.6084/m9.figshare.19210806 ^6^

This project contains the following underlying data:

Data file 1. (Description of data.)

Data file 2. (Description of data.)

Data are available under the terms of the Creative Commons Zero “No rights reserved” data waiver Attribution 4.0 International (CC BY 4.0).

## Author contributions

All author: writing-original draft; BS and FF: conceptualization and Resources

## Ethical approval

The study was approved by the Ethics Committee (Approval number 0340/LOE/301.4.2/II/2021) of Dr Soetomo Academic Medical Center.
