# Supplementary material for: Differences in the length of postoperative care of endoscopic endonasal surgery in simple and complex surgical procedure groups
Source: F1000Res. 2022 Sep 2;11:619. Originally published 2022 Jun 7. [Version 2] doi: 10.12688/f1000research.110037.2 (PMC9758441; doi:10.12688/f1000research.110037.2)
Supplement: Peer review history

# Peer review: Reviewer response for version 2

**DOI:** 10.5256/f1000research.137567.r149290

**Published:** 2022-12-16

**Authors:** Mikiya Asako

**Affiliations:** 1 Department of Otolaryngology − Head and Neck Surgery, Kansai Medical University Hospital Medical Center, Osaka, Japan

I approved Dr Suktikno B revision version. I think this paper is acceptable.

Is the work clearly and accurately presented and does it cite the current literature?

Yes

If applicable, is the statistical analysis and its interpretation appropriate?

Yes

Are all the source data underlying the results available to ensure full reproducibility?

Yes

Is the study design appropriate and is the work technically sound?

Partly

Are the conclusions drawn adequately supported by the results?

Partly

Are sufficient details of methods and analysis provided to allow replication by others?

Yes

Reviewer Expertise:

Rhinology, Endoscopic Surgery, Skull base Surgery, Rhinoplasty, Allegology

I confirm that I have read this submission and believe that I have an appropriate level of expertise to confirm that it is of an acceptable scientific standard.

# Peer review: Reviewer response for version 2

**DOI:** 10.5256/f1000research.137567.r149291

**Published:** 2022-09-16

**Authors:** Kachorn Seresirikachorn

**Affiliations:** 1 Department of Otolaryngology, Faculty of Medicine, Chulalongkorn University, Bangkok, Thailand

Thank you for addressing my comment.

Is the work clearly and accurately presented and does it cite the current literature?

Partly

If applicable, is the statistical analysis and its interpretation appropriate?

Partly

Are all the source data underlying the results available to ensure full reproducibility?

Yes

Is the study design appropriate and is the work technically sound?

Partly

Are the conclusions drawn adequately supported by the results?

Partly

Are sufficient details of methods and analysis provided to allow replication by others?

Partly

Reviewer Expertise:

Chronic rhinosinusitis, allergic rhinitis, endoscopic sinus and skull base surgery

I confirm that I have read this submission and believe that I have an appropriate level of expertise to confirm that it is of an acceptable scientific standard.

# Response

**Published:** 2022-09-16

**Authors:** Budi Sutikno

**Affiliations:** World Hearing Forum, Indonesia

Dear Reviewer (Dr. Kachorn),

 Thank you very much for your kind assistance to this manuscript.

# Peer review: Reviewer response for version 1

**DOI:** 10.5256/f1000research.121607.r144481

**Published:** 2022-08-01

**Authors:** Kachorn Seresirikachorn

**Affiliations:** 1 Department of Otolaryngology, Faculty of Medicine, Chulalongkorn University, Bangkok, Thailand

I have a question about the rationale for performing this study. The surgery for the different diseases should not be compared. This paper's simple endoscopic endonasal surgery is the surgery for allergic rhinitis or unilateral maxillary sinusitis, whereas complex endoscopic endonasal surgery is the surgery for true sinus disease. The pathophysiology of the disease is different, so I am not convinced that these two categories of surgery should be compared.

 The definition of postoperative care should be provided. I am not sure that the postoperative care period in this paper means only the debridement and sinonasal toilet or the entire postoperative management, including all medical treatment to normalize the sinonasal mucosa.

 Postoperative wound healing depends on the surgical technique and the surgeon's expertise. The qualification and standard technique of the surgeon should be provided. Moreover, I am not sure that all patients receive the same postoperative protocol. This point is essential when the objective aim to compare the two surgical categories.

 The clinical and research applicability and the limitation of the study should be discussed.

Is the work clearly and accurately presented and does it cite the current literature?

Partly

If applicable, is the statistical analysis and its interpretation appropriate?

Partly

Are all the source data underlying the results available to ensure full reproducibility?

Yes

Is the study design appropriate and is the work technically sound?

Partly

Are the conclusions drawn adequately supported by the results?

Partly

Are sufficient details of methods and analysis provided to allow replication by others?

Partly

Reviewer Expertise:

Chronic rhinosinusitis, allergic rhinitis, endoscopic sinus and skull base surgery

I confirm that I have read this submission and believe that I have an appropriate level of expertise to confirm that it is of an acceptable scientific standard, however I have significant reservations, as outlined above.

# Response

**Published:** 2022-08-14

**Authors:** Budi Sutikno

**Affiliations:** World Hearing Forum, Indonesia

Dear Reviewer (dr. Kachorn)

 Thank you very much for the kind review on my manuscript. This study was intended to give brief difference of post operative care duration for different group procedure which represent group of disease: localized and diffuse mucosal disease. I hope it will be beneficial to clinicians to give advice for their patients on how long the post operative care will take. 

 I would like to have your further review on the revised manuscript I have sent to the publisher.

# Peer review: Reviewer response for version 1

**DOI:** 10.5256/f1000research.121607.r139884

**Published:** 2022-06-24

**Authors:** Mikiya Asako

**Affiliations:** 1 Department of Otolaryngology − Head and Neck Surgery, Kansai Medical University Hospital Medical Center, Osaka, Japan

This paper describes appropriate messages for rhinologists of the postoperative care. Their opinion is clear and correct. However, I still have a small unclarified issue about the indication of surgery. I think the inflammation of the complex group is even worse than the simple group. The different profiles of airway inflammation strongly involve results. The simple group might be including non-type 2 CRS or allergic rhinitis, on the other hand, the complex group will be including type2 CRS, because CRS w NP is predominant in central compartments, such as ethmoid and frontal sinus. Type 2 CRS is much more severe inflammatory changes in the epithelial membrane. Please explain the differences of postoperative damage as well as the profiles of airway inflammation.

Is the work clearly and accurately presented and does it cite the current literature?

Yes

If applicable, is the statistical analysis and its interpretation appropriate?

Yes

Are all the source data underlying the results available to ensure full reproducibility?

Yes

Is the study design appropriate and is the work technically sound?

Partly

Are the conclusions drawn adequately supported by the results?

Partly

Are sufficient details of methods and analysis provided to allow replication by others?

Yes

Reviewer Expertise:

Rhinology, Endoscopic Surgery, Skull base Surgery, Rhinoplasty, Allegology

I confirm that I have read this submission and believe that I have an appropriate level of expertise to confirm that it is of an acceptable scientific standard, however I have significant reservations, as outlined above.

# Response

**Published:** 2022-07-01

**Authors:** Budi Sutikno

**Affiliations:** World Hearing Forum, Indonesia

Dear Prof. Mikiya Asako,

 Thank you for kind review. Please allow me to give short explanation for your question. Due to limitation of resources, we lay the endotyping on post operative histopathological report. Most of CRSwNP had non-type 2 endotype and pathological result showed neutrophilic predominant inflammatory polyps. Some cases easily (not so easy) managed with topical intranasal steroid and nasal saline irrigation, but some other difficult cases need systemic treatment of low dose long term macrolide. 

 Looking forward to having your further respons. Thank you very much.

# Response

**Published:** 2022-08-14

**Authors:** Budi Sutikno

**Affiliations:** World Hearing Forum, Indonesia

Dear Reviewer (dr. Asako)

 I have send the revised manuscript with additional information on the simple and complex group. You are right that simple group represent the localized disease, whereas the complex group represent the diffuse mucosal disease. In regard with the revised manuscript, I would like to have your further review. Thank you very much in advance.
